# The Removal of Stains from the Teeth, Etc.

**Published:** 1885-05

**Authors:** A. W. Harlan

**Affiliations:** Chicago, Ill.


### SELECTIONS.

### The Removal of Stains From the Teeth Caused by the Administration of Medicinal Agents and the Bleaching of Pulpless Teeth.

BY A. W. HARLAN, M.D., CHICAGO, ILL.

Read in the Section of Oral and Dental Surgery of the American Medical Association, May, 1884.

GENELEMEN:—A large number of remedial agents administered by physicians temporarily stain the teeth, but in looking over the list I find there are but few which may be said to permanently stain them. The mineral acids—nitric, sulphuric, hydrochloric, and other acids of this nature, if used for any length of time, may discolor the teeth and likewise have a deleterious effect on them; yet it cannot be said that such agents stain the teeth so that any particular method should be desired for restoring their natural appearance. The vegetable series may likewise be dismissed. The tannates and astringents generally do not permanently stain the teeth. The muriated tincture of iron, and in fact all the ferrum preparations, with the single exception of ferrum dialystum, do stain the teeth; yet it is comparatively easy to remove such stain, by the use of dentifrices and tooth pastes, when of recent occurrence. Occasionally it may be necessary to polish the teeth with wooden or leather points charged with finely pulverized emery or levigated pumice; following these, powdered Arkansas stone and precipitated chalk, incorporated with a thick solution of white castile soap, should be used. The stains produced by hydrastis, aloes, tobacco, rhubarb, ink, infusion of saffron, pinus canadensis, carmine, catechu, hydriodic acid, tincture of iodine, and kindred agents, are of temporary duration in nearly all cases. Not so with tobacco, when the enamel has been fractured, or the masticating surfaces of teeth have been deprived of their enamel by wear. In such cases there is no remedy for the stain, when it disfigures, except to cap the teeth with gold. This last conditon it is seldom found necessary to treat, as in such cases generally it is only imperative when the ends of the teeth have become sensitive to thermal change. Staining of the teeth as a result of age, it is not the province of this paper to treat. The single substance, which I have found to stain the teeth permanently, is nitrate of silver. Solutions are frequently used in the mouth and throat of too great strength. Many times the powdered nitrate is used to dust diseased mucous membranes, and the teeth then suffer. Occasionally, dental surgeons use the solid stick on the walls of sensitive teeth, and also to arrest incipient caries. When carelessly used it may stain other teeth than those operated upon. These cases are the ones we are called upon to treat. It matters not how the stains are acquired, they must be removed, and no amount of polishing, without injury to the external surfaces of the teeth, will remove such stains. The agents which may be used for removing such stains are not numerous. Cyanide of potassium, iodide of potassium, tincture of iodine, and the liq. amm. fortior are all recommended. There are other substances which may be used to remove fresh stains, notably solution of chloride of sodium, if used immediately. I recommend as the neatest, safest, and most certain method, the adjustment of the rubber dam; then dry the teeth, and paint three or four of them with the compound tincture of iodine, allowing it to dry; then moisten the surfaces of the teeth with the stronger liquor ammonia for two or three minutes, and wash the surfaces with peroxide of hydrogen. The stains will have disappeared in consequence of the chemical change having taken place resulting in the formation of iodide of silver and nitrate of ammonia. The teeth should then be polished in the usual manner.

BLEACHING TEETH.

It is not intended that a history of the various methods of bleaching teeth in use should be presented for consideration at this time; but it is necessary to remark that most of them are faulty and very few useful in all cases. In order to bleach a pulpless tooth the operator must first fill the root at least one-third its length. All decay should be removed and the fragments of the pulp in the fine angles of the outline of the living pulp should also be removed. Discolored dentine if hard need not be cut away. With the rubber dam adjusted over the adjacent teeth, including the one to be operated upon, the cavity is thoroughly washed with $H_2$ $O_2$ repeatedly and then carefully dried by using the hot blast from a powerful bulb syringe. A small quantity of choride of alumina is placed within the cavity, and it is moistened with peroxide of hydrogen and allowed to remain five minutes. The deliquesced $Al_2$ $Cl_6$ is carefully washed out of the cavity with a clear solution of sodæ biboras Na. 2, $B_4$ $O_7$-10 $H_2$ O, and the cavity thoroughly dessicated. In the vast majority of cases the tooth will have returned to its normal color. The cause of the change in color is owing to the complete oxidation of the infiltrate into the tubules and the destruction of the staining of the contents of the tubules. The tooth should not be bathed in creosote, carbolic acid, alcohol, or any other substance capable of coagulating albumen. Where the operator has reason to suspect that any such agent has been introduced into the cavity, it should first be washed with the clear solution of sodæ biboras, and then followed by the above-mentioned method. The rapid liberation of chlorine from $Al_2$ $Cl_6$ in the presence of $H_2$ $O_2$, resulting in the formation of H-Cl and $H_2$ O, leaving unsatisfied O and Cl accounts for the speedy destruction of the coloring matters within the pulpless tooth. In order to maintain the color an oxy-chloride of suitable color should be used to fill the remainder of the pulp canal, and as large a portion of unfilled cavity as judgment will indicate; thirty to fifty minutes should be given for the hardening of the oxy-chloride. The cavity should be filled with gold immediately. It is not wise to allow saliva or other liquids to come in contact with the oxy-chloride after it is introduced into the tooth. It jeopardizes the permanency of every case when oxy-chloride becomes moist, on account of the speedy absorption of fluids by the freshly hardened filling.

De Lavra (*El Siglo Medico*) gave extract of gelseminum in a case of refractory sciatica in a woman eight months pregnant. He gave three doses of one grain each. The cure was rapid and permanent.

